# Multidrug-Resistant Stenotrophomonas maltophilia Keratitis in a Penetrating Keratoplasty Patient

**DOI:** 10.7759/cureus.20412

**Published:** 2021-12-14

**Authors:** Nikhil Thakral, Fraser S Peck, Pantelis Ioannidis

**Affiliations:** 1 Family Medicine, Trevelyan House Surgery, London, GBR; 2 Pharmaceutical Medicine, Richmond Pharmacology, London, GBR; 3 Ophthalmology, Eastbourne District General Hospital, Eastbourne, GBR

**Keywords:** multidrug-resistant, microbial keratitis, multidrug-resistant pathogen, keratoplasty, bacterial keratitis, maltophilia, stenotrophomonas

## Abstract

We describe the management of a case of multidrug-resistant *Stenotrophomonas maltophilia* in a patient who had previously undergone photorefractive keratectomy and subsequent penetrating keratoplasty for Schnyder’s crystalline corneal dystrophy. This pathogen is rare and, in this case, was multi-drug resistant.

## Introduction

This article was presented as an e-poster at the 2021 Scottish Clinical Fellow Conference on November 10, 2021.

Bacterial keratitis is a significant corneal pathology linked to corneal scarring and is associated with up to 5% of global blindness [1]. Factors that can increase the risk of infectious keratitis include contact lens wear, ocular surface diseases, corneal surgery and trauma [2].

An example of such an ocular surface disease is Schnyder’s crystalline corneal dystrophy. This is an autosomal dominant disease characterised by bilateral progressive corneal opacification leading to reduced visual acuity and glare. Penetrating keratoplasty is the management of choice to recover vision quality [3].

Accurate, early diagnosis of the causative organism in bacterial keratitis is key as it allows for targeted antimicrobial therapy based on sensitivities [4]. *Strenotrophomonas maltophilia* is a gram-negative bacillus that has also been implicated as a hospital-acquired respiratory pathogen [5]. *S. maltophilia* has previously been implicated in bacterial keratitis [6].

## Case presentation

A 77-year-old female patient presented to ophthalmic services with a 10-day history of blurred vision in her left eye. There is a past medical history of Schnyder’s crystalline corneal dystrophy. This was treated with photorefractive keratectomy 10 years previously in the affected eye and then a penetrating keratoplasty two years previously in the affected eye. The patient also has primary open-angle glaucoma. There was no concurrent contact lens usage. Treatment at presentation included loteprednol etabonate 0.5%, latanoprost 0.005% and timolol 0.5% eye drops.

**Figure 1:**
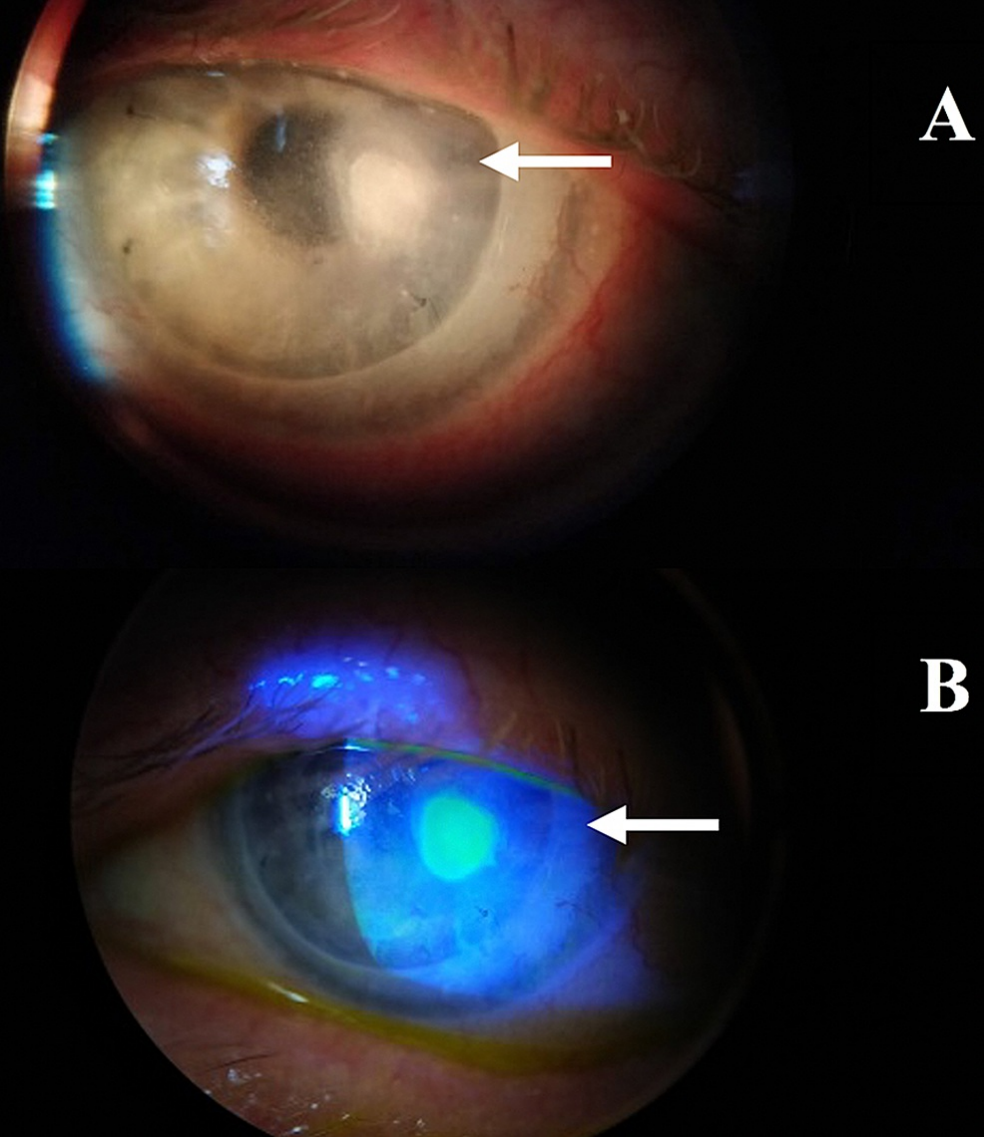
(A) Slit-lamp biomicroscopy of the S. maltophilia ulcer in the left eye (see top arrow). (B) Fluorescein staining shows the epithelial defect of the ulcer (see bottom arrow).

On examination, her pinhole visual acuity in the left eye was 6/24 and rebound tonometry of 9mm Hg. There was a 2 by 2mm epithelial defect with epithelial oedema, mild stromal haze and hyperaemia (Figures 1A, 1B), there was no anterior chamber reaction.

A corneal scrape was conducted, a culture was taken and used for MALDI-TOF analysis. The organism was identified as *S. maltophilia* (Table 1) showing multidrug resistance. Initial management with an intensive course of preservative-free cefuroxime 5% and gentamicin 1.5% reducing with time, also loteprednol etabonate 0.5% was stopped.

**Table 1:** The antibiotic sensitivities of the corneal scrape isolate

Antibiotic	Sensitivity
Amikacin	Resistant
Amoxicillin	Resistant
Aztreonam	Resistant
Cefotaxime	Resistant
Cefuroxime	Resistant
Chloramphenicol	Resistant
Ciprofloxacin	Resistant
Co-Amoxiclav	Resistant
Co-Trimoxazole	SENSITIVE
Cefpodoxime	Resistant
Ertapenem	Resistant
Gentamicin	Resistant
Piperacillin-tazobactam	Resistant
Trimethoprim	Resistant

Following sensitivities, oral Co-Trimoxazole, 960mg, twice a day was initiated and given for three weeks. Cefuroxime 5% drops were changed to preservative-free levofloxacin 0.5% on a reducing course. One week later, the epithelium defect had lessened to 1 by 2mm, with mild anterior chamber activity and keratic precipitates. At this point, hydrocortisone 0.335%, two drops, four times a day was initiated.

Following an eight-week treatment regimen, a small stable corneal scar remains. The corneal graft is clear with no signs of rejection and pinhole visual acuity of 6/18 is observed in the left eye. Lubricating eye drops have been initiated with dexamethasone 0.1% once daily to encourage scar remodulation.

## Discussion

A key element in ocular infections caused by *S. maltophilia* is ocular surface instability [7]. Corneal epithelial cells secrete surfactant proteins which can inhibit microbial infection. In cases where the barrier of corneal epithelial cells is compromised, there is a reduction in the intrinsic antimicrobial properties of the tear film. This can facilitate the growth of opportunistic infections such as *S. maltophilia* [8]. In this case, the previous history of Schnyder’s crystalline corneal dystrophy, photorefractive keratectomy, penetrating keratoplasty and the use of loteprednol etabonate were likely risk factors by altering the natural resistance to infection [2]. 

This case highlights a relatively uncommon pathogen affecting the eye. Its most frequent manifestations are pneumonia and bacteremia [5]. There is reported literature that implicates this organism as a cause of bacterial keratitis [8]. In one case series, *S. maltophilia* was isolated from polybacterial ocular infections in 10 out of 15 cases suggesting this pathogen may be more common than previously thought [6]. This case also highlights the use of oral Co-Trimoxazole as there was no commercial ophthalmic preparation available.

*S. maltophilia* has intrinsic microbial resistance. This includes an impermeable outer membrane which makes it resistant to most antibiotics [9] as well as the production of two beta-lactamases (L1 and L2), conferring resistance to beta-lactam-containing antibiotics [10]. Multidrug-resistant bacteria are a growing global concern and are considered a major public health problem [11]. 

A recent case series recommended high bacterial sensitivity to fluoroquinolones (93%), gentamicin (88%) and beta-lactam (81%) classes of antibiotics. It also suggests that concurrent use of fluoroquinolone, beta-lactam and an aminoglycoside should be considered first-line treatment [8]. In this case, the isolated strain of S.maltophilia showed multidrug resistance to commonly prescribed topical antibiotics and was only sensitive to Co-Trimoxazole.

## Conclusions

*S. maltophilia* as a cause of bacterial keratitis is uncommon. It is also apparent that concerns regarding the management of multi-drug resistant pathogens are growing and it is a significant public health problem. In cases where there is no commercial ophthalmic preparation for an antibiotic, oral antibiotics can be used. Prompt and accurate diagnosis of the causative organism helps to reduce the risk of prolonged infection, reducing permanent damage to vital ocular tissues and subsequent morbidity.
